# Supplementary material for: Development of a computer-aided design software for the quantitative evaluation of aesthetic damage
Source: PLoS One. 2019 Dec 18;14(12):e0226322. doi: 10.1371/journal.pone.0226322 (PMC6919621; doi:10.1371/journal.pone.0226322)
Supplement: S1 Code site — 1. (ZIP) [file pone.0226322.s001.zip › [EESC_jr][Projeto]Relatorio_07_21/reports/templates_zip/gentelella-master.zip!/gentelella-master/vendors/DateJS/reports/lcov-report/core/time_period.js.html]

Code coverage report for core/time\_period.js


# Code coverage report for core/time\_period.js

## Statements: 33.33% (21 / 63)      Branches: 2.7% (1 / 37)      Functions: 36.36% (4 / 11)      Lines: 33.33% (21 / 63)      Ignored: none

All files » core/ » time\_period.js

```
|  |  |  |
| --- | --- | --- |
| 1 2 3 4 5 6 7 8 9 10 11 12 13 14 15 16 17 18 19 20 21 22 23 24 25 26 27 28 29 30 31 32 33 34 35 36 37 38 39 40 41 42 43 44 45 46 47 48 49 50 51 52 53 54 55 56 57 58 59 60 61 62 63 64 65 66 67 68 69 70 71 72 73 74 75 76 77 78 79 80 81 82 83 84 85 86 87 88 89 90 91 92 93 94 95 96 97 98 99 100 101 | 1   1 1 7         1 7         1 1 7 7 7 7       1 1                                           1                       1                                               1 1                   1   1   1 | ``` (function () { 	"use strict"; 	var attrs = ["years", "months", "days", "hours", "minutes", "seconds", "milliseconds"]; 	var gFn = function (attr) { 		return function () { 			return this[attr]; 		}; 	}; 	 	var sFn = function (attr) { 		return function (val) { 			this[attr] = val; 			return this; 		}; 	}; 	var addSetFuncs = function (context, attrs) { 		for (var i = 0; i < attrs.length ; i++) { 			var $a = attrs[i], $b = $a.slice(0, 1).toUpperCase() + $a.slice(1); 			context.prototype[$a] = 0; 			context.prototype["get" + $b] = gFn($a); 			context.prototype["set" + $b] = sFn($a); 		} 	};   	var setMonthsAndYears = function (orient, d1, d2, context) { 		function inc() { 			d1.addMonths(-orient); 			context.months++; 			if (context.months === 12) { 				context.years++; 				context.months = 0; 			} 		} 		if (orient === +1) { 			while (d1 > d2) { 				inc(); 			} 		} else { 			while (d1 < d2) { 				inc(); 			} 		} 		context.months--; 		context.months *= orient; 		context.years *= orient; 	};   	var adjustForDST = function(orient, startDate, endDate) { 		var hasDSTMismatch = (false === (startDate.isDaylightSavingTime() === endDate.isDaylightSavingTime())); 		if (hasDSTMismatch && orient === 1) { 			startDate.addHours(-1); 		} else if (hasDSTMismatch) { 			startDate.addHours(1); 		} 	}; 	/** 	 * TimePeriod(startDate, endDate); 	 * TimePeriod(years, months, days, hours, minutes, seconds, milliseconds); 	 */ 	var TimePeriod = function (years, months, days, hours, minutes, seconds, milliseconds) { 		if (arguments.length === 7) { 			this.set(years, months, days, hours, minutes, seconds, milliseconds); 		} else if (arguments.length === 2 && arguments[0] instanceof Date && arguments[1] instanceof Date) { 			var startDate = arguments[0].clone(); 			var endDate = arguments[1].clone(); 			var orient = (startDate > endDate) ? +1 : -1; 			this.dates = { 				start: arguments[0].clone(), 				end: arguments[1].clone() 			};   			setMonthsAndYears(orient, startDate, endDate, this); 			adjustForDST(orient, startDate, endDate); 			// // TODO - adjust for DST 			var diff = endDate - startDate; 			if (diff !== 0) { 				var ts = new TimeSpan(diff); 				this.set(this.years, this.months, ts.getDays(), ts.getHours(), ts.getMinutes(), ts.getSeconds(), ts.getMilliseconds()); 			} 		} 		return this; 	}; 	// create all the set functions. 	addSetFuncs(TimePeriod, attrs); 	TimePeriod.prototype.set = function (years, months, days, hours, minutes, seconds, milliseconds){ 		this.setYears(years \|\| this.getYears()); 		this.setMonths(months \|\| this.getMonths()); 		this.setDays(days \|\| this.getDays()); 		this.setHours(hours \|\| this.getHours()); 		this.setMinutes(minutes \|\| this.getMinutes()); 		this.setSeconds(seconds \|\| this.getSeconds()); 		this.setMilliseconds(milliseconds \|\| this.getMilliseconds()); 	};   	Date.TimePeriod = TimePeriod;   	Eif (typeof window !== "undefined") { 		// keeping API compatible for v1.x  		window.TimePeriod = TimePeriod; 	} }()); ``` |
```

Generated by istanbul at Sun Nov 16 2014 12:08:33 GMT-0800 (PST)
